# Effects of trimethylamine *N*-oxide and urea on DNA duplex and G-quadruplex

**DOI:** 10.1080/14686996.2016.1243000

**Published:** 2016-11-16

**Authors:** Yu-mi Ueda, Yu-ki Zouzumi, Atsushi Maruyama, Shu-ichi Nakano, Naoki Sugimoto, Daisuke Miyoshi

**Affiliations:** ^a^ Faculty of Frontiers of Innovative Research in Science and Technology (FIRST), Konan University, Kobe, Japan; ^b^ Department of Biomolecular Engineering, Graduate School of Bioscience and Biotechnology, Tokyo Institute of Technology, Yokohama, Japan; ^c^ Frontier Institute for Biomolecular Engineering Research (FIBER), Konan University, Kobe, Japan

**Keywords:** DNA, G-quadruplex, duplex, molecular crowding, osomolyte, 30 Bio-inspired and biomedical materials, 505 Optical/Molecular spectroscopy, 208 Sensors and actuators, 101 Self-assembly/Self-organized materials

## Abstract

We systematically investigated effects of molecular crowding with trimethylamine *N*-oxide (TMAO) as a zwitterionic and protective osmolyte and urea as a nonionic denaturing osmolyte on conformation and thermodynamics of the canonical DNA duplex and the non-canonical DNA G-quadruplex. It was found that TMAO and urea stabilized and destabilized, respectively, the G-quadruplex. On the other hand, these osmolytes generally destabilize the duplex; however, it was observed that osmolytes having the trimethylamine group stabilized the duplex at the lower concentrations because of a direct binding to a groove of the duplex. These results are useful not only to predict DNA structures and their thermodynamics under physiological environments in living cells, but also design of polymers and materials to regulate structure and stability of DNA sequences.

## Graphical abstract

**Figure:**
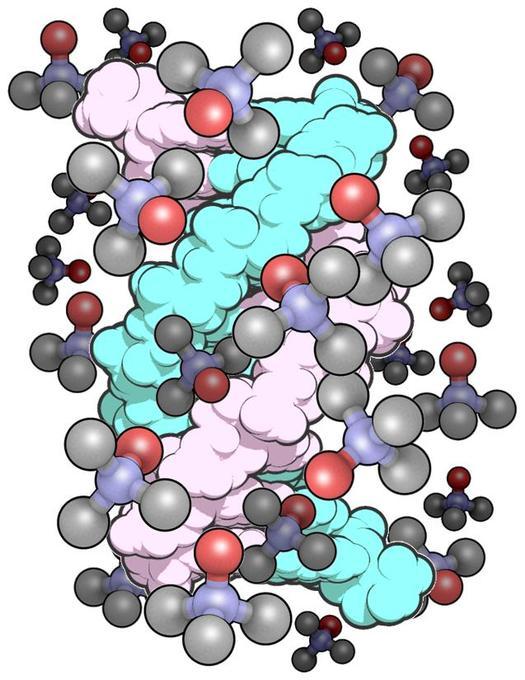

## 1. Introduction

Molecular crowding largely affects equilibrium and rate of macromolecular interactions and reactions.[1–3] Understanding the molecular crowding effects on property and function of biomolecules is one of the important topics in broad fields of research from biology to chemistry. The molecular crowding conditions observed in living cells arise not only from macromolecules such as proteins, nucleic acids, and polysaccharides but also small osmotic metabolites such as amino acids, methylamines, monosaccharides, polyols, and urea.[4–7] Most of these osmolytes protect biomolecules and stabilizes biomolecular structure.[7] On the other hand, urea destabilizes structures and reduces functions of biomolecules. These effect of osmolytes are additive such that they canceled each other. A methylamine osmolyte, trimethylamine *N*-oxide (TMAO), is well known as a counteracting osmolyte, and has an ability to protect proteins from denaturation induced by urea.[8,9] The most effective counteraction at a 2:1 urea:TMAO ratio is similar to their ratio observed in living cells.[10–12] Therefore, the effects of TMAO and urea on properties of biomolecules have drawn the attention of many researchers including chemists, biologist, and pharmacologists.

There are number of reports studying how osmolytes alter structure and thermal stability of nucleic acids to compare with their effects on proteins. Although *N*,*N*,*N*-trimethylammonioacetate (GB; glycine betaine) and TMAO counterpart the denaturation of proteins by urea,[8,9,13,14] it was demonstrated that GB destabilizes DNA duplexes [15–17] as shown in urea. It has also been demonstrated that various osmolytes including GB and TMAO destabilize RNA secondary (hairpin loop) structure [18–20] but osmolyte stabilizes and destabilizes RNA tertiary structures, depending on cation and osmolyte species, base sequence, and temperature.[21,22] Therefore, general osmolyte effects and interactions on proteins have not been extensively tested with nucleic acids. Moreover, there are few systematic reports for molecular crowding effects with naturally occurring osmolytes on non-canonical DNA secondary structures, such as a three-stranded triplex and a four-stranded quadruplex.

**Figure 1.**
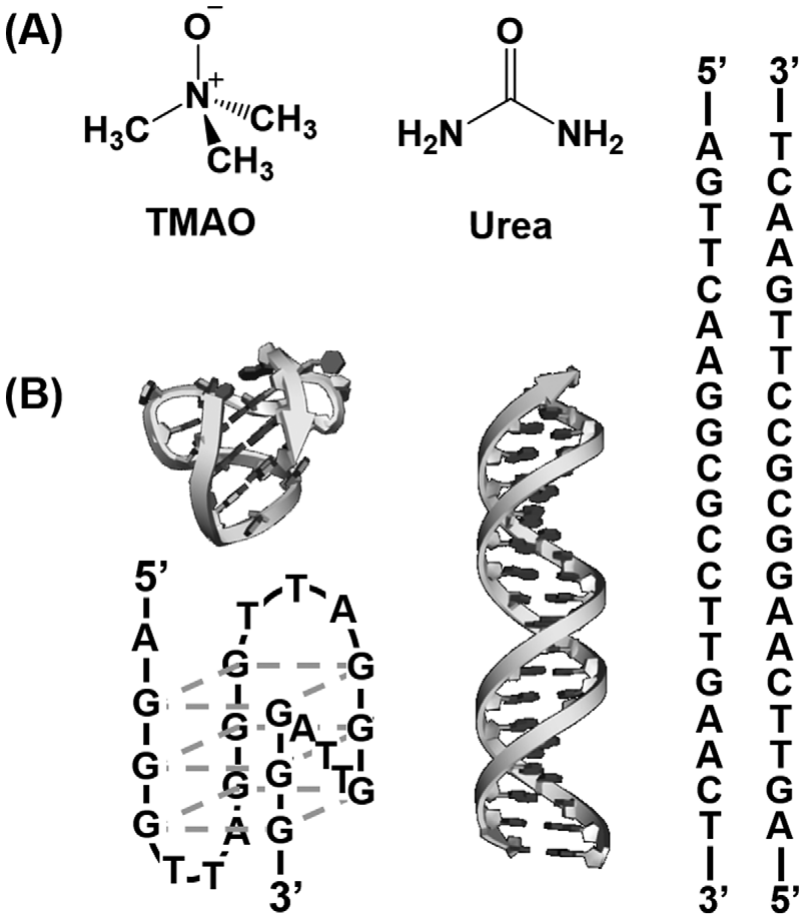
(A) Chemical structures of TMAO and urea. (B) Schematic illustrations and sequences of gqDNA (left) forming the G-quadruplex and dsDNA (right) forming the duplex.

In this study, we systematically investigated effects of molecular crowding with TMAO as a zwitterionic and protective osmolyte and urea as a nonionic denaturing osmolyte on conformation and thermodynamics of a DNA duplex as the canonical structure, and a DNA G-quadruplex as the non-canonical DNA structure (Figure 1). Conformational analysis of the DNA oligonucleotides showed that the osmolytes used in this study did not alter structure of the DNA oligonucleotides. Thermodynamic parameters of the DNA duplex showed that the urea monotonically destabilized the duplex, whereas the TMAO effect on the DNA duplex was dependent on its concentration; lower concentrations of TMAO stabilized the duplex and higher concentrations led to destabilization. On the other hand, urea and TMAO destabilized and stabilized, respectively, the G-quadruplex. These results demonstrated for the first time how naturally occurring osmolytes affect the canonical and non-canonical DNA structures. Moreover, it was suggested that effects of molecular crowding with the osmolytes largely depended not only on the DNA secondary structure, but also osmolyte species inducing molecular crowding conditions. Quantitative comparisons of molecular crowding effects induced by other small molecules further indicated that a trimethylamine group involved in molecular crowding reagents played an important role in determining how molecular crowding reagents, including osmolytes, affect thermodynamics of DNA secondary structures.

## 2. Materials and methods

### 2.1. Materials

All the high-pressure liquid chromatography (HPLC)-grade DNA strands used in this study were from Sigma-Aldrich Japan K. K. (Tokyo, Japan) and Hokkaido System Science Co., Ltd (Hokkaido, Japan). Concentration of the strands was adjusted as described previously.[24,25] Chemical reagents of reagent grade were purchased from Wako Pure Chemical Co., Ltd (Osaka, Japan) or Sigma-Aldrich Japan K. K.

### 2.2. CD spectroscopy

Circular dichroism (CD) spectra were obtained with a spectropolarimeter (J-820, JASCO Co., Ltd, Hachioji, Japan) in a buffer containing 100 mM KCl, 10 mM K_2_HPO_4_ (pH 7.0) and EDTA-2 K at 25 °C. The DNA samples (10 μM) were refolded by cooling from 90 °C to 25 °C at a rate of 0.5 °C min^−1^. The temperature of the cuvette was controlled by a temperature controller (PTC-348, JASCO Co., Ltd).

### 2.3. Thermodynamic analysis

The thermal melting curve of the DNA samples was obtained by monitoring the absorption at 260 nm for the duplex and 295 nm for the G-quadruplex by use of a UV-1800 spectrometer (Shimadzu Co., Ltd, Kyoto, Japan) equipped with a temperature controller. The melting curves were obtained in the 100 mM KCl buffer. The DNA samples were refolded by cooling from 93 to 0 °C at a rate of 0.5 °C min^−1^. The samples were then heated from 0 to 93 °C at 0.5 °C min^−1^ to trace the thermal denaturation curves. The thermodynamic parameters for the DNA structural formation were calculated by a curve fitting procedure as described previously.[26,27] It should be noted that a two-state assumption of the DNA structural denaturation and renaturation was required for the evaluation of the thermodynamic parameters.

### 2.4. Water activity calculations

The activity of water molecules was measured by use of a pressure osmometer (520XR, Wescor, Logan, UT, USA) based on the osmotic stress method.[28] All the measurements were carried out at room temperature.

## 3. Results and discussion

### 3.1. Structural analysis of DNA strands

The purpose of this study is to quantify the effects of osmolytes on conformation and thermodynamics of the non-canonical DNA structures as well as the canonical DNA duplex (Figure 1). The four-stranded G-quadruplex was used as the non-canonical structure. A 22-mer DNA strand, gqDNA [dA(GGGTTA)_3_GGG], which was derived from the human telomere sequence, was used as a G-quadruplex-forming DNA sequence.[23] We also designed a DNA sequence (dsDNA: [dAGTTCAAGGCGCCTTGAACT]) to form a self-complementary canonical duplex.[29] As osmolytes, we focused on a zwitterionic TMAO, and a nonionic urea, both of which are typical osmolytes existing in living organisms and have been studied for their effects on biomolecules, especially proteins.

**Figure 2.**
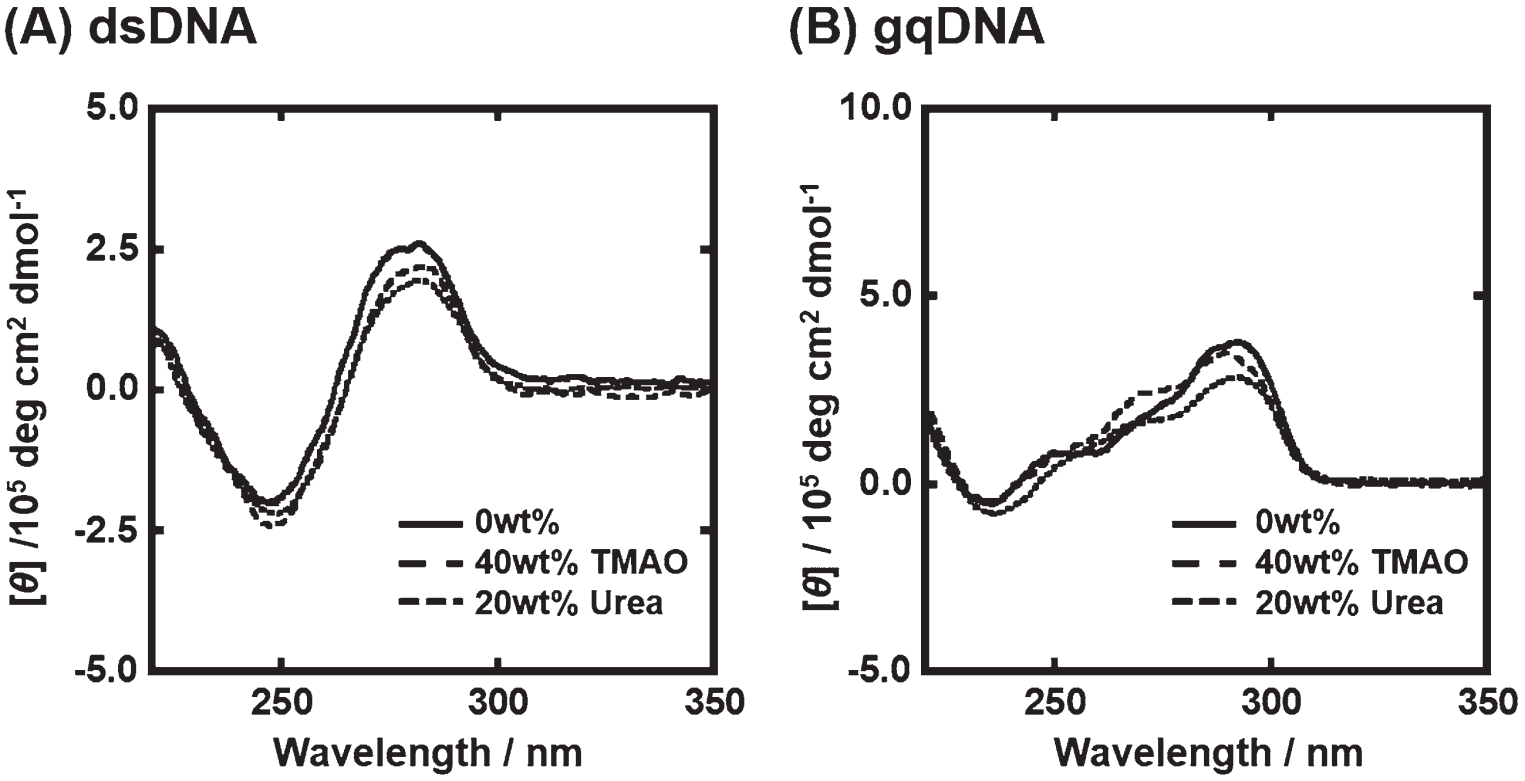
CD spectra of 10 μM dsDNA (A) and gqDNA (B) without osmolyte (continuous line), with 40wt% TMAO (broken line) or with 20wt% urea (dotted line). All measurements were carried out in the buffer containing 100 mM KCl, 10 mM K_2_HPO_4_, and 1 mM K_2_EDTA at 25 °C.

Since the experimental condition largely affects non-canonical DNA structures and their thermodynamics,[30] we first studied the structure of gqDNA and dsDNA in the absence and the presence of osmolytes. Figure 2(A) shows the CD spectra of 10 μM dsDNA in the buffer of 100 mM KCl in the absence and in the presence of osmolyte at 25 °C. In the absence of osmolyte, the CD spectrum of dsDNA showed positive and negative peaks around 260 nm and 245 nm, respectively, indicating B-form duplex of dsDNA.[31] CD spectra of dsDNA in the presence of TMAO or urea were almost identical with one in the absence of osmolyte, also showing the B-form duplex formation. Figure 2(B) shows CD spectra of gqDNA in the same experimental conditions. The CD spectra showed large and small positive peaks around 295 nm and 260 nm, respectively, which is typical for a mixed G-quadruplex,[23,32] in which only one guanine-rich tract is in the opposite direction from other three tracts (Figure 1(B)). This mixed G-quadruplex structure found in this study is consistent with the solution structure in the presence of K^+^ studied by nuclear magnetic resonance.[23] These results show that the osmolytes do not largely affect conformation of the DNA sequences.

### 3.2. Osmolyte effects on thermal stability of the DNA structures

**Figure 3.**
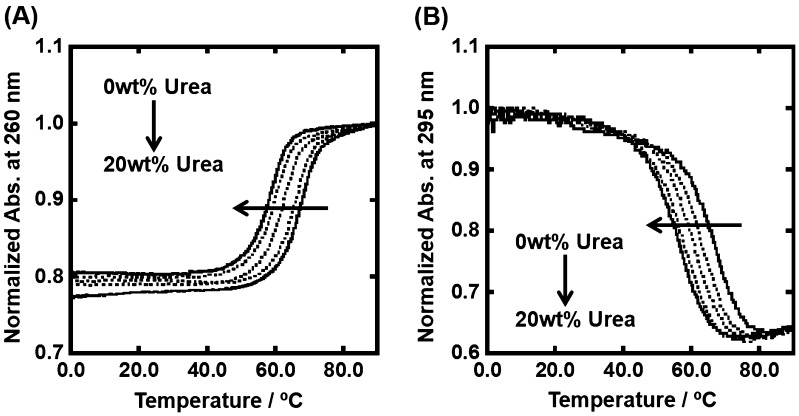
Normalized UV melting curves for 5 μM dsDNA (A) and gqDNA (B) in the buffer containing 100 mM KCl, 10 mM K_2_HPO_4_, and 1 mM K_2_EDTA with various concentrations of urea (0, 5, 10, 15, and 20wt%). UV melting curves of dsDNA and gqDNA were assessed by UV absorbance at 260 nm and 295 nm, respectively.

Figure 3(A) shows UV melting curves traced at 260 nm of 5 μM dsDNA in the 100 mM KCl buffer in the presence of 0, 5, 10, and 20 wt% urea. As urea is a typical denaturant for biomolecules, the value of melting temperature (*T*_m_) of the duplex decreased from 66.4 °C to 58.0 °C. Figure 3(B) shows UV melting curves traced at 295 nm of 5 μM dsDNA in the same experimental conditions. The *T*_m_ value of the G-quadruplex decreased from 66.5 °C to 55.7 °C. Table 1 shows thermodynamic parameters (Δ*G*º_37_, Δ*H*º, Δ*S*º) for formation of the duplex and G-quadruplex in the presence of various concentrations of TMAO or urea. The values of Δ*G*º_37_ further showed quantitatively that urea monotonically destabilized the duplex and G-quadruplex. In contrast to the destabilization effect of urea, the effect of molecular crowding with TMAO on the duplex was nonlinear. The addition of TMAO from 0 wt% to 20 wt% stabilized the duplex from Δ*G*º_37_ = –17.3 kcal mol^–1^ to be –18.6 kcal mol^–1^, whereas further addition of 40 wt% TMAO destabilized to be Δ*G*º_37_ = –16.0 kcal mol^–1^. The addition of 20 wt% TMAO stabilized monotonically the G-quadruplex from –5.0 kcal mol^–1^ to be –9.5 kcal mol^–1^. The G-quadruplex of gqDNA was too stable to perfectly denature even at 95 °C in the presence of higher concentrations of TMAO (data not shown). Although thermodynamic parameters were not able to be evaluated, destabilization of the G-quadruplex was not observed with the higher concentrations of TMAO. Moreover, the thermodynamic parameters show that the TMAO effects result from the enthalpic change, which has a larger effect than the entropic change, although the thermodynamic parameters of the duplex in the presence of urea is an exception. These enthalpic-dominant contributions are consistent with previous reports for the effects of nonionic cosolutes on the thermodynamics of the DNA structures, such as duplex, triplex, and G-quadruplex, and suggest that hydration of the DNA structures is one of critical factors for determining the thermal and thermodynamic stability of the DNA structures.[33,34] On the other hand, the destabilization of the duplex by urea was dominated by unfavorable entropic change, which exceeds favorable enthalpic change. This may correspond with the denaturant property of urea by inhibition of hydrogen bindings between base pairs stabilizing the duplex or by disruption of the structure of water molecules.[35] Despite extensive studies, it is still unclear how urea denatures DNA structures, which we attempted to clarify in this study.

**Table 1.** Thermodynamic parameters for the structural formation of dsDNA and gqDNA in the presence of various concentrations of urea or TMAO.

DNA	[Urea] (wt%)	ΔGº37 (kcal mol^–1^)	ΔH (kcal mol^–1^)	ΔS (cal) mol–1 K^–1^	Tm^a^(°C)
*In the presence of urea*					
dsDNA	0	−17.3	−113	−307	66.4
					
	5	−17.0	−115	−317	64.9
	10	−16.3	−119	−331	61.7
	15	−15.8	−123	−345	59.5
	20	−14.6	−112	−313	58.0
gqDNA	0	−5.15	−59.2	−174	66.5
	5	−4.44	−57.2	−170	63.1
	10	−3.62	−50.8	−152	60.8
	15	−2.70	−42.3	−128	58.2
	20	−2.66	−46.9	−143	55.7
*In the presence of TMAO*					
dsDNA	0	−17.3	−113	−307	66.4
	5	−18.0	−117	−319	67.4
	10	−18.5	−117	−319	69.0
	15	−18.4	−116	−316	69.0
	20	−18.6	−116	−315	69.6
	30	−17.9	−111	−300	68.9
	40	−16.0	−96.8	−261	66.8
gqDNA	0	−5.15	−59.2	−174	66.5
	5	−6.24	−62.1	−180	71.6
	10	−7.08	−66.2	−191	74.1
	15	−8.03	−70.1	−200	77.1
	20	−9.50	−78.6	−223	79.7

^a^ The *T*_m_ values were evaluated with 5 μM DNA concentration.

### 3.3. Hydration change through formation of the DNA structures

Since the thermodynamic parameters suggest that hydration of the DNA structures is one of the important factors determining how the osmolytes affect the thermodynamics of the DNA structure, we further attempted to evaluate the number of water molecules released or taken up upon formation of the DNA structures. The folding of the DNA structure proceeds in a solution containing water molecules (H_2_O), osmolytes (OS), and potassium ions (K^+^) can be represented as the following:[36]

(1) $${\text{DNA}}_{\text{unfolding}\;}⇄{\;\text{DNA}}_{\text{folding}}\;\text{+}\;\Delta n_{\text{w}}H_{2}\text{O}\;\;\text{+}\;\;\Delta n_{\text{os}}\text{OS}+\Delta n_{K^{+}}K_{+}$$

where Δ*n*_w_, Δ*n*_os_, Δ*n*_K+_ are the numbers of water molecules, osmolytes, and potassium ion, respectively, released through folding of the DNA structures. In this equilibrium at a constant temperature and pressure, the relationship between the true thermodynamic equilibrium constant (*K*_0_) and the observed one (*K*_obs_) that is $K_{0}=K_{\text{obs}}a_{w}^{\Delta n_{\text{w}}}a_{\text{os}}^{\Delta n_{\text{os}}}a_{{\text{K}}^{+}}^{\Delta n_{{\text{K}}^{+}}}$ can be represented by Equation (2).[36]

(2) $$\frac{d\ln K_{\text{obs}}}{d\ln a_{\text{w}}}\;\text{=}\;\;-\left[\Delta n_{\text{w}}\;\;\text{+}\;\;\Delta n_{\text{os}}\left(\frac{d\ln a_{\text{os}}}{d\ln a_{\text{w}}}\right)\;\;\text{+}\;\;\Delta n_{{\text{K}}^{+}}\left(\frac{d\ln a_{\text{K}+}}{d\ln a_{\text{w}}}\right)\right]$$

where *a*_w_, *a*_os_, and *a*_K+_ are the activities of water molecules, osmolytes, and potassium ions, respectively.

**Figure 4.**
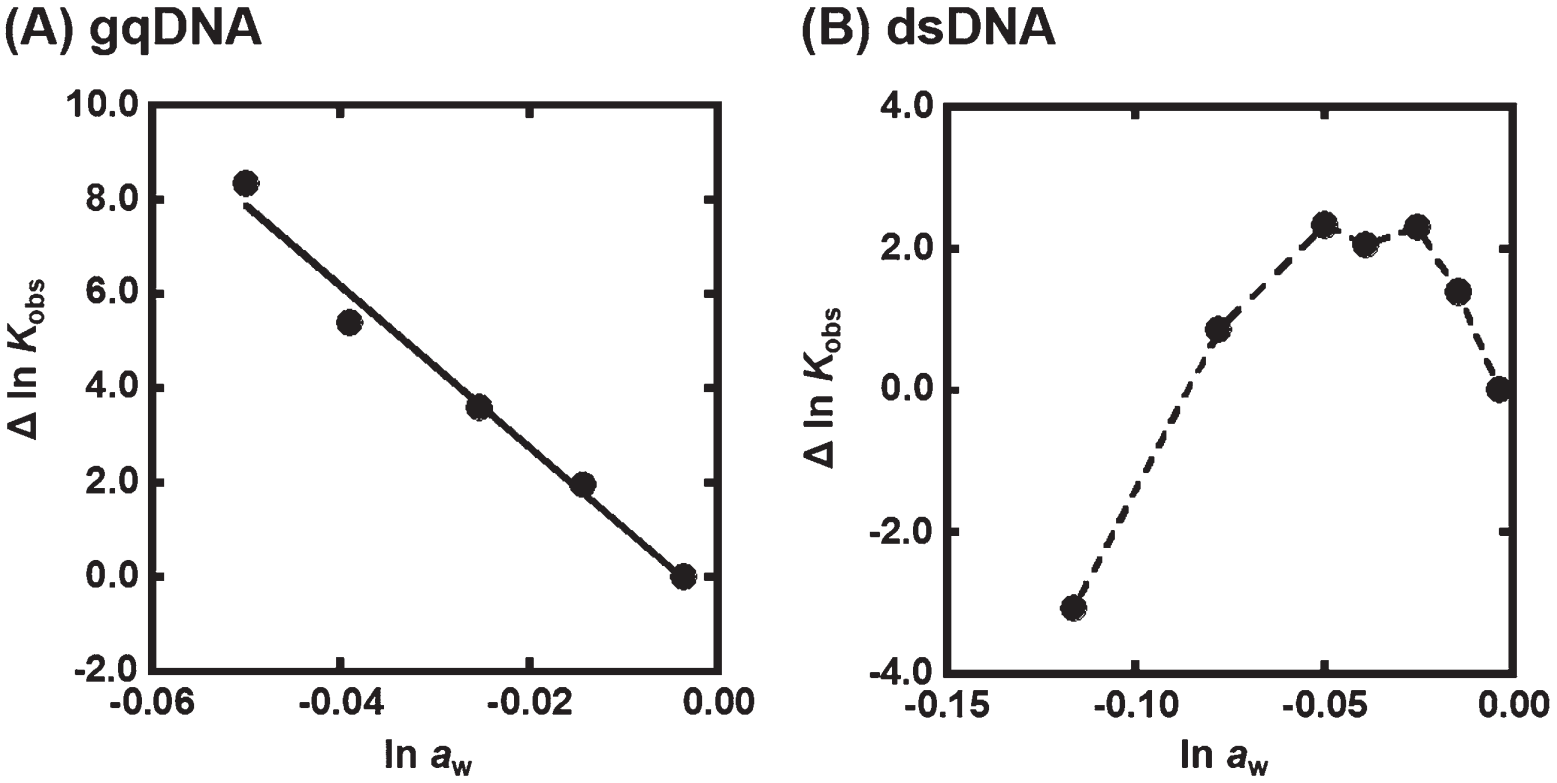
(A) Δln *K*_obs_ vs. ln *a*_w_ plots for the formation of the gqDNA in the buffer containing 100 mM KCl, 10 mM K_2_HPO_4_, and 1 mM K_2_EDTA with various concentrations of TMAO (0, 5, 10, 15, and 20wt%). (B) Δln *K*_obs_ vs. ln *a*_w_ plots for the formation of the gqDNA in the buffer containing 100 mM KCl, 10 mM K_2_HPO_4_, and 1 mM K_2_EDTA with various concentrations of TMAO (0, 5, 10, 15, 20, 30, and 40wt%).

**Figure 5.**
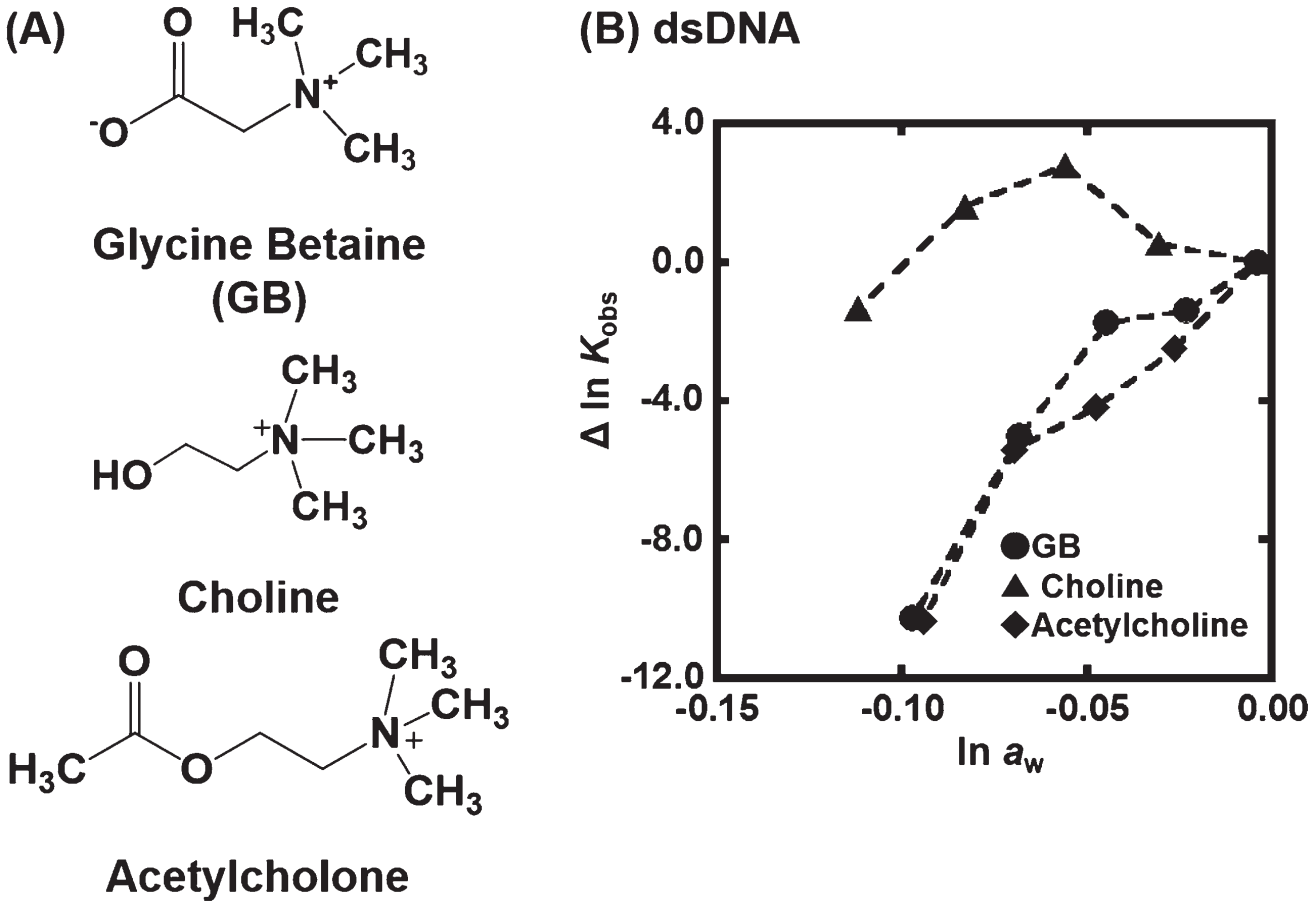
(A) Chemical structure of glycine betaine (GB), choline, and acetylcholine. (B) Δln *K*_obs_ vs. ln *a*_w_ plots for the formation of the dsDNA in the buffer containing 100 mM KCl, 10 mM K_2_HPO_4_, and 1 mM K_2_EDTA with various concentrations of GB (circles), choline (rectangles), or acetylcholine (diamonds) (0, 10, 20, 30, and 40wt%).

Figure 4 shows the plots of Δln *K*_obs_ {Δln *K*_obs_ = ln *K*_obs_ (in the presence of osmolyte) – ln *K*_obs_(in the absence of osmolyte)} vs. ln *a*_w_ in the presence of various concentrations of TMAO at 25 °C. The values of ln *a*_w_ can be obtained by osmotic pressure measurements. The G-quadruplex of gqDNA showed a linear relationship between Δln *K*_obs_ and ln *a*_w_ (Figure 4(A)). Thus, the value of Δ*n*_w_ was evaluated to be +170 from the slope, corresponding to 170 water molecules per structure (7.7 water molecules per nucleotide) released upon the G-quadruplex formation. In the case of the dsDNA (Figure 4(B)), the plot did not show a linear relationship but a convex shape. This nonlinear relationship indicates that not only hydration but also other factors are important for the TMAO effects on the duplex. We have demonstrated a linear relationship between ln *K*_obs_ and ln *a*_w_ for various nucleic acid structures with various molecular crowding with nonionic reagents.[30,33,34] This is the first example showing a nonlinear relationship between ln *K*_obs_ and ln *a*_w_. There are two possible differences between the nonionic reagents [glycerol, ethylene glycol, and poly(ethylene glycol), etc., which we used in previous studies] and the zwitterionic TMAO; the zwitterionic TMAO has positive and negative functional groups, although TMAO is charge neutral. Another difference is that TMAO has a trimethylamine (Figure 1) group, which has been reported to bind grooves of the DNA duplex.[37,38] From this point of view, as molecular crowding reagents we utilized other small molecules, zwitterionic GB, and positive 2-hydroxy-*N*,*N*,*N*-trimethylethanamonium (choline) and 2-acetoxy-*N*,*N*,*N*-trimethylethanaminium (acetylcholine), having the trimethylamine group (Figure 5(A)). Figure 5(B) shows the plots of Δln *K*_obs_ vs. ln *a*_w_ with various concentrations of these molecular crowding reagents as well as TMAO at 25 °C. All plots show convex shapes, although degree of increment of *K*_obs_ depends on molecular crowding reagents. These results suggest that the trimethylamine group leads to the nonlinear relationship.

### 3.4. Factors affecting the molecular crowding effects

It was previously reported that A–T base pairs are more stable than G–C base pairs in the hydrated ionic liquid including choline as a cationic component,[39] and found that choline ions stabilized A–T-rich DNA duplexes by binding in the minor of a DNA duplex.[40] Moreover, alkylammonium ions such as tetramethylammonium stabilizes A–T-rich duplexes by preferentially binding to the minor groove of A–T base pairs.[41,42] These results are consistent with the findings here that the molecular crowding reagents having the trimethylamine group stabilize the duplex of dsDNA in lower concentrations. Higher concentrations of these molecular crowding reagents lead to destabilization of the duplex. A reduction of activity of water molecules by addition of molecular crowding reagents is unfavorable for formation of the DNA duplex because water molecules are taken up upon duplex formation.[30,36] In addition, the stabilization effect of choline is larger than others. In the previous report, it was demonstrated by molecular dynamics simulations that choline was able to bind A–T base pairs through formation of a hydrogen bond.[40] Thus, the largest stabilization effect of choline on the duplex found in this study is qualitatively consistent with the molecular dynamics simulations.

**Figure 6.**
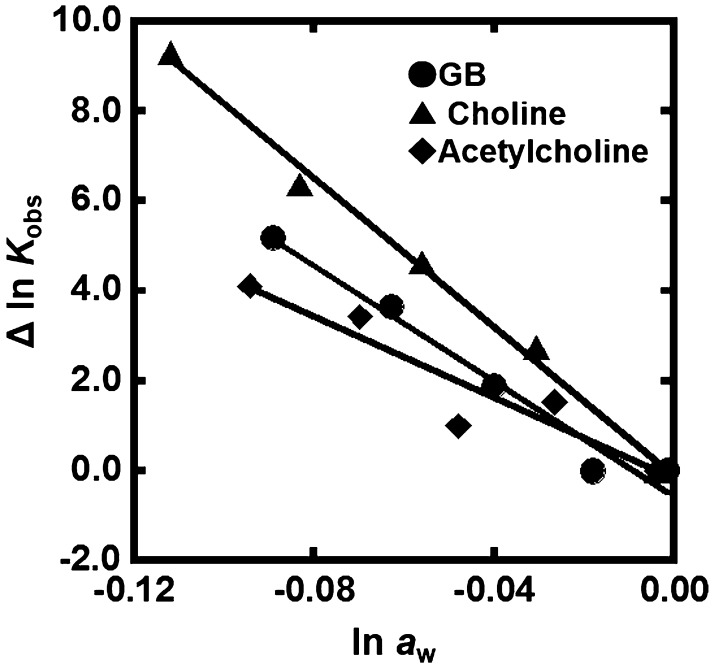
Δln *K*_obs_ vs. ln *a*_w_ plots for the formation of the gqDNA in the buffer containing 100 mM KCl, 10 mM K_2_HPO_4_, and 1 mM K_2_EDTA with various concentrations of GB (circles), choline (rectangles), or acetylcholine (diamonds) (0, 10, 20, 30, and 40wt%).

Figure 6 shows the plots of Δln *K*_obs_ of the G-quadruplex of gqDNA vs. ln *a*_w_ with various concentrations of GB, choline, and acetylcholine. As shown for TMAO, a linear relationship was observed. From the slopes of the linear relationship, we evaluated the values of Δ*n*_w_ for GB, choline, and acetylcholine to be +83, +64, and +45, respectively. Although these positive values show dehydration of the G-quadruplex upon folding, the values depend on the molecular crowding reagents, indicating direct effects of molecular crowding reagents on the thermodynamics of the G-quadruplex. This dependency observed in the G-quadruplex also supports the direct binding of the molecular crowding reagents to the DNA structures.

## 4. Conclusions

In this study, we systematically investigated how TMAO and urea affect thermodynamics of DNA secondary structures. It was found that TMAO and urea stabilized and destabilized, respectively, the G-quadruplex. On the other hand, these osmolytes generally destabilize the duplex. More importantly, it was observed that osmolytes and molecular crowding reagents having a trimethylamine group stabilized the duplex of dsDNA at lower concentrations. From these results, it was suggested that the trimethylamine group played a critical role in the stabilization of the DNA duplex. These results are useful not only for predicting DNA structures and their thermodynamics under physiological environments in living cells, but also for designing polymers and materials to regulate structure and stability of DNA sequences.

## Disclosure statement

No potential conflict of interest was reported by the authors.

## Funding

This work was supported by Grants-in-Aid for Scientific Research [KAKENHI 15H03840, 16K14042] from MEXT, Japan, by Scientific Research on Innovative Areas ‘Nanomedicine Molecular Science’ [number 2306] and ‘Strategic Research Foundation at Private Universities’ from JSPS, Japan, and by the Asahi Glass Foundation.
